# Application of cell-free DNA for genomic tumor profiling: a feasibility study

**DOI:** 10.18632/oncotarget.26642

**Published:** 2019-02-15

**Authors:** Lise B. Ahlborn, Kristoffer S. Rohrberg, Migle Gabrielaite, Ida V. Tuxen, Christina W. Yde, Iben Spanggaard, Eric Santoni-Rugiu, Finn C. Nielsen, Ulrik Lassen, Morten Mau-Sorensen, Olga Østrup

**Affiliations:** ^1^ The Phase I Unit, Department of Oncology, Rigshospitalet, Copenhagen University, Copenhagen, Denmark; ^2^ Center for Genomic Medicine, Rigshospitalet, Copenhagen University, Copenhagen, Denmark; ^3^ Department of Pathology, Rigshospitalet, Copenhagen University, Copenhagen, Denmark

**Keywords:** cfDNA, liquid biopsy, genomic profile, biopsy, WES

## Abstract

**Purpose:**

Access to genomic tumor material is required to select patients for targeted therapies. However, tissue biopsies are not always feasible and therefore circulating cell-free DNA (cfDNA) has emerged as an alternative. Here we investigate the utility of cfDNA for genomic tumor profiling in the phase I setting.

**Study design:**

Peripheral blood was collected from patients with advanced solid cancers eligible for phase I treatment. Patients failing the initial tissue biopsy due to inaccessible lesions or insufficient tumor cellularity (<10%) were included in the study. Genomic profiling of cfDNA including whole exome sequencing (WES) and somatic copy number alterations (SCNAs) analysis (OncoScan).

**Results:**

Plasma cfDNA was pro- and retrospectively profiled from 24 and 20 patients, respectively. The median turnaround time was 29 days (*N*= 24, range 13-87 days) compared to tissue re-analyses of median 60 days (*N*= 6, range 29-98). Selected cancer-associated alterations (SCAAs) were identified in 70% (31/44) of patients, predominantly by WES due to the low sensitivity of OncoScan on cfDNA. Primarily, inaccessible cases of prostate and lung cancers could benefit from cfDNA profiling. In contrast, breast cancer patients showed a low level of tumor-specific cfDNA which might be due to cancer type and/or active treatment at the time of plasma collection.

**Conclusion:**

Plasma cfDNA profiling using WES is feasible within a clinically relevant timeframe and represents an alternative to invasive tissue biopsies to identify possible treatment targets. Especially, difficult-to-biopsy cancers can benefit from cfDNA profiling, but tumor tissue remains the gold standard for molecular analyses.

## INTRODUCTION

Knowledge of the genomic makeup of tumors is essential for cancer diagnosis, prognosis, and selection of treatment. With the great advancements in the field of precision medicine, genotype-directed therapy is becoming a standard tool for stratification of oncological patients. For instance, *HER2* gene expression and *EGFR* mutation status are used to guide treatment for breast and non-small cell lung cancers (NSCLC), respectively [1, 2]. Ideally, fresh tissue is used to characterize the tumor but often archival material such as formalin-fixed paraffin-embedded (FFPE) tissue is used introducing several problems. First, archival tissue might not represent the current malignancy due to clonal evolution of the disease over time and in response to previous therapies [3, 4]. Second, DNA from FFPE tissue are often highly fragmented influencing downstream analyses [5, 6]. Although tissue biopsies, either fresh or archival, represent standard for molecular testing, poor quality or inadequate quantity of tissue and DNA is often challenging besides the discomfort and risks of complications related to biopsy procedures. In NSCLC, tissue biopsies are unusable in 20-30% of patients [7], highlighting the need for an alternative source of tumor material.

Circulating cell-free DNA (cfDNA) has been widely investigated as a potential surrogate for tissue biopsies for non-invasive assessment of tumor-related genomic alterations as circulating tumor DNA (ctDNA) can be identified in cfDNA. Recently, FDA approved *EGFR*-mutation testing based on cfDNA for treatment stratification of NSCLC [8]. Due to the rapid development of NGS technology, it is now possible to characterize the molecular profile of cfDNA [9, 10] but no previous studies have investigated comprehensive cfDNA profiling in a prospective setting. The focus of whole exome sequencing (WES) studies has been on characterizing tumor heterogeneity and resistance [11, 12] and only few prospective studies have included cfDNA analyses, focusing mainly on treatment monitoring using small gene-panels [13]. In a prospective study, Kaisaki *et al*., showed that targeted-sequencing of cfDNA in early stage lung cancers, was a valuable alternative to tissue in a diagnostic setting due to high concordance of tumor mutations between tissue and cfDNA [14]. Little has been reported on turnaround time of cfDNA analyses in a prospective setting, but results from retrospective cohorts have reported analysis times between 10-15 days [10, 15, 16]. Furthermore, studies characterizing somatic copy number alterations (SCNAs) in cfDNA have been sparse [17].

We performed a feasibility study including a pro- and retrospective cohort of patients eligible for phase I treatment where tissue was inaccessible for biopsy or the biopsy was too low in tumor cell content. Genomic cfDNA profiling included WES and SCNAs analyses (OncoScan). The aim of the study, was to investigate whether tumor-specific DNA alterations could be identified in plasma cfDNA and whether the analysis could be performed within a time frame relevant in a clinical setting.

## RESULTS

### Patient characteristics

**Figure 1:**
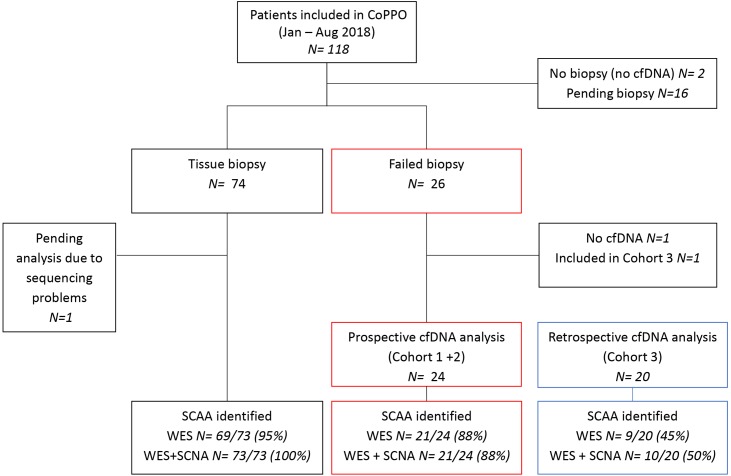
Genomic profiling of the CoPPO cohort within the study period A total number of 118 patients were included in the CoPPO project from January to 1^st^ of August 2018. Eighteen patients were excluded because no plasma was collected for cfDNA analysis or tumor biopsies were pending. Tissue biopsies were usable for genomic profiling in 74 cases and a SCCA (selected cancer-associated aberrations) were identified in 95% and 100% of patients when using WES (whole exome sequencing) or WES+SCNA (somatic copy number alteration) analysis, respectively. The gray/red boxes indicate 26 patients with failed tissue biopsies either due to low tumor cell content (<10%, Cohort 2) or to inaccessible tumors (Cohort 1). Two patients were excluded; one due to lack of cfDNA and the other was included in Cohort 3 due prolonged start of the cfDNA-pipeline due to implementation of the setup in the laboratory. Twenty-four patients were prospectively profiled based on plasma cfDNA and a SCAA was identified using WES alone or plus SCNA analysis in 88% of the patients. In the retrospective cohort (gray/blue boxes) a SCAA was found in 45% using WES and in 50% when OncoScan analysis was included. *N* indicate the number of patients in each group.

A total of 118 advanced cancer patients were prospectively enrolled in the CoPPO project from January to August 2018 (Figure 1). Of these, 24 patients (17%) underwent cfDNA analysis either because the tumor tissue was inaccessible for biopsy (*N*=9, Cohort 1) or the obtained tissue biopsy had low tumor cellularity (*N*= 15, Cohort 2). Various cancer types were included, the most represented being colorectal (*N*=5) and prostate cancer (*N*=4) (Table 1). Six patients had subsequently a successful re-biopsy performed and three cases had archival FFPE tissue analyzed. Twenty patients were included in a retrospective cohort (Cohort 3), selected from the total CoPPO cohort as illustrated in Supplementary Figure 1. Eight patients from Cohort 3 were re-biopsied and genomic reports on tumor tissue were obtained. Across cohorts, an equal distribution of males (50%) and females (50%) were included and 41% (18/44) of the patients received treatment at the time of plasma collection (Supplementary Table 1). Furthermore, most patients had multiple metastatic sites with the most common biopsy site being liver, representing 67% (22/33) of the biopsy sites (Supplementary Table 1). Radiological assessment of the overall tumor burden was not available at the time of plasma sampling. Assessment of overall tumor burden is not a standard procedure in the CoPPO study and would not impact the enrollment of patients.

**Table 1:** Patient characteristics

Characteristic	Prospective Cohorts 1+2 (*N*= 24)	Retrospective Cohort 3 (*N*= 20)	All patients (*N*= 44)
**Age (median, range)**	62 (36 – 82)	64 (26 – 75)	64 (26 – 82)
**Gender**			
Male	12	10	22 (50%)
Female	12	10	22 (50%)
**Tumor origin**			
Colorectal	5	10	15
Breast	3	5	8
Prostate	4	1	5
Endometrial	2	0	2
Head and neck	1	1	2
Bile duct	1	2	3
Lung (NSCLC)	3	0	3
Ovarian	1	0	1
Other^A^	4	1	5
**Cohorts**			
1 (Prospective, no biopsy)	9	-	-
2 (Prospective, failed biopsy)	15	-	-
3 (Retrospective)	-	20	-
**Number of patients with ≥ 1 SCAA^B^**	21 (88%)	10 (50%)	31 (70%)
**Turnaround time (days)**			
cfDNA, *N*= 24 (median, range)	29 (13-87)	-	-
Tissue, *N*= 6 (median, range)	60 (29-98)	-	-
**Profiling on other materials**			
Tissue re-biopsy	6	8	14
Archival FFPE tissue	3	0	3

^A^Included: Mesothelioma *N*=1, Testicular *N*=1, Pancreatic *N*=1, Gastric *N*= 1, Cervical *N*=1

^B^SCAA: Selected cancer-associated alterations

### Feasibility of genomic cfDNA profiling

#### Method efficacy

Cell-free DNA was successfully extracted from all prospectively enrolled patients with concentrations ranging from 1.5 to 120 ng/ml plasma (median 8.0 ng/ml) (Supplementary Table 2). The retrospective cohort was selected based on available cfDNA (Supplementary Figure 1) with concentrations from 2.2 to 181.5 ng/ml plasma (median 26.2 ng/ml) (Supplementary Table 2). Whole exome sequencing was performed on all patients (*N*=44) with a median overall average coverage of 186x (interquartile range IQR=273; Q1=90.80; Q3=364.30) and median 10x coverage on 94% of the exome (interquartile range IQR=0.0135; Q1=0.9344; Q3=0.9479) (Supplementary Figure 2 and Supplementary Table 2), similar to previous studies [18, 19].

OncoScan array used varying cfDNA input concentrations (≥ 5 ng) depending on the amount remaining after WES analysis (Supplementary Table 2). The median input was 20 ng cfDNA (range 7-80 ng). The OncoScan analysis was achieved on 40 out of 44 patients, as four samples had insufficient input material for full genomic profiling and therefore WES was prioritized. Of the 40 samples, 8 samples failed the analysis due to quality issues of either cfDNA (*N*= 1, P42) or OncoScan array (*N*= 7, “Failed (suboptimal quality)” in Supplementary Table 4). Noticeably, cfDNA concentration or input amount did not seem to affect ctDNA detection by OncoScan or WES although the small sample size could influence this result (*P*>0.05, t-test, unequal variance).

#### Turnaround time

The median turnaround time for cfDNA profiling in Cohort 1 and 2 was 29 days (range 13-87 days), defined as the median time from failed tissue biopsy until the genomic report was completed (Supplementary Figure 3). The analysis time decreased and stabilized during the study due to improvement and establishment of the cfDNA workup. A fresh tissue re-biopsy was obtained and successfully profiled for six patients in the prospective cohorts. The median turnaround time was 60 days (range 29-98) defined as the time from failed biopsy until completion of the genomic report on the re-biopsy (Supplementary Figure 3). Of note, inclusion of patients in Cohort 1, where no biopsy could be obtained, commenced after the initial tests of the cfDNA pipeline.

### Detection of genomic tumor alterations in cfDNA

**Figure 2:**
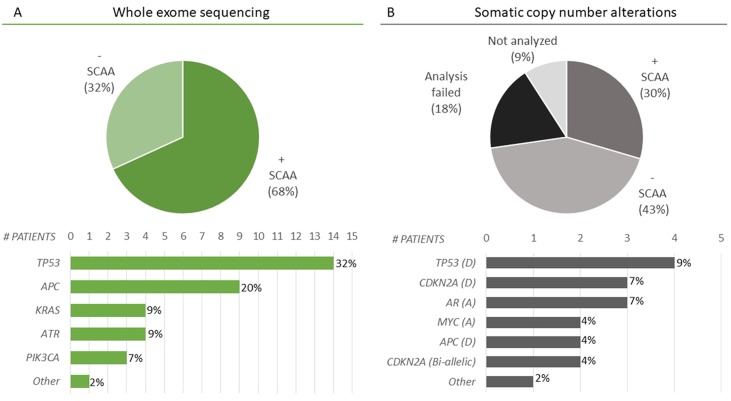
Selected cancer-associated alterations (SCAAs) identified in cfDNA **(A)** WES was successfully performed on all cfDNA samples (*N*= 44). SCAAs were identified in 68% (30/44) of patients (+ SCAA). The most frequently altered genes were *TP53, APC, KRAS, ATR*, and *PIK3CA*. Furthermore, 30 different genes (*NRAS, PIK3CG, BRAF, ATM, ARID1A, AKT2, FGF10, PCDHB12, ACIN1, RAD21, RAD51C, MYO6, SMARCA5, RAB14, FANCD2, CHD7, PTEN, AR, MAP3K9, WT1, POLR3B, MAP2K2, HDAC9, SOX9, MAP2K, SMAD4, RAD50, SMARCC2, CHEK1, IDH1)* where mutated in only a single patient each, indicated by *Other*. Information on the individual alterations are included in Supplementary Table 3. **(B)** Analysis of somatic copy number alterations (SCNA) by OncoScan identified SCAAs in 30% of patients (13/44) most often involving deletion (*D*) or amplification (*A*) of *TP53* (*N=* 4)*, CDKN2A* (*N=* 3)*, AR* (*N=* 3)*, MYC* (*N=* 2), or *APC* (*N=* 2). *Other* included genes that were mutated in only a single patient being: *CCND1 (A), KRAS (A), JAK2 (A), MET (A), PTEN (bi-allelic).* The bar plot, include only genes where a SCNA was identified together with a mutation in the same gene leading to both alleles affected. All SCNAs are reported in Supplementary Table 4. A silent chromosomal profile (- SCAA) was found in 43% (19/44) of patients. The analysis failed in 18% (8/44) of the cases due to suboptimal quality of the cfDNA (*N*=1) or arrays (*N*=7). In 4 cases (9%), the amount of cfDNA was insufficient for analysis.

**Figure 3:**
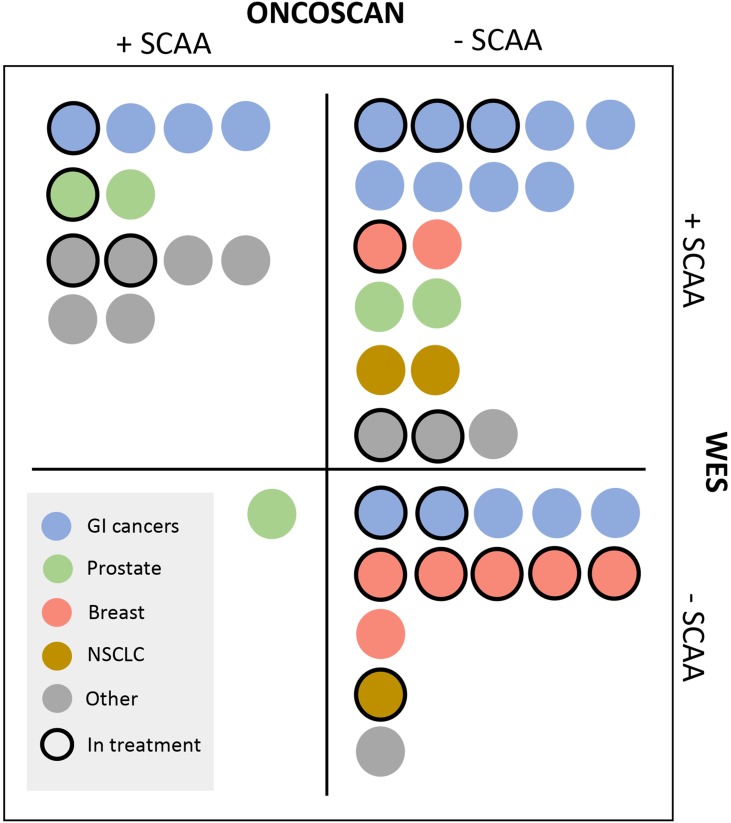
OncoScan and WES results indicated by cancer type and active treatment (*N*=44) Identification of a selected cancer-associated alteration (SCAA) from either OncoScan or WES is indicated by *+SCAA*. Negative reports from WES (*no SCAA*, Supplementary Table 3) and *Silent profiles*, *Failed*, plus *No analysis* from OncoScan analyses (Supplementary Table 4) are indicated as *-SCAA*. Cancer types represented ≥3 times across cohorts, are color coded as shown in the legend. Gastrointestinal (GI) cancers included bile duct (*N*=3) and colorectal cancers (*N*=15). Cancer types “Other” included: Endometrial *N*=2, Gastric *N* =1, Ovarian *N* =1, Head and neck *N* =2, Mesothelioma *N* =1, Testicular *N* =1, Pancreatic *N* =1, Cervical *N* =1. Patients in active treatment at the time of plasma cfDNA collection are marked with a full-line border. Additional individual information is provided in Supplementary Table 1, 3, and 4.

A complete genomic cfDNA report including both WES and OncoScan (+/- SCAA) was achieved in 32/44 cases (Figure 2). Whole exome sequencing was successfully performed in all 44 cases and at least one SCAA was identified in 68% (30/44 patients) of patients. The most frequently altered genes were *TP53* (*N*=14), *APC* (*N*=9), *KRAS* (*N*=4), *ATR* (*N*=14) and *PIK3CA* (*N*=3) (Figure 2A and Supplementary Table 3). A range of 30 different SCAAs was observed only once (*N*=1). In 32% (14/44 patients), WES did not detect a SCAA with almost half of these being breast cancers including a large fraction of patients in active treatment at the time of blood collection (Figure 3). Significantly more breast cancers were observed in the -SCAA group compared to the +SCAA group from either WES or OncoScan (*P*= 0.0048, Firsher's exact test), whereas the fractions in treatment were not significantly different between the two SCAA-groups (*P*= 0.098, Firsher's exact test). OncoScan array was successfully performed on 32/44 samples with acceptable quality values, with 43% (*N*=19) showing a silent chromosomal profile and 30% (*N*=13/44) with detectable SCAAs (Figure 2B and Supplementary Table 4). The most common SCAA were deletion of either *TP53* (*N*=4), *CDKN2A* (*N*=3), or *APC* (*N*=2) and amplification of *AR* (*N*=3) and *MYC* (*N*=2). Furthermore, bi-allelic loss of *CDKN2A* was observed in two patients (*N*=2). As previously mentioned, 18% (*N*=8) of the samples failed the analyses and 9% (*N*=4) were not analyzed due to limited cfDNA material (Figure 2B).

A SCAA was identified from either WES or OncoScan in 70% of the patients (31/44) involving all cancer types included in the study (Figure 3). This was lower than the overall CoPPO cohort (100%, Figure 1), however expectable from the type of input material. Whole exome sequencing lead to detection of SCAAs in 95% of the tissue biopsies compared to 88% and 45% in cfDNA from the pro-and retrospective cohorts, respectively (Figure 1). Adding the OncoScan SCNA analysis to the cfDNA profiling did not increase the number of positive findings in the prospective cohorts and only included one more patient (P35, prostate cancer with *AR* amplification) in the retrospective cohort.

### Comparing genomic profiles identified by WES in plasma and tissue DNA

Fourteen re-biopsies and three archival FFPE samples were included in the study to compare the tumor tissue to cfDNA (total *N*= 17). Nine of the sample-pairs were obtained in the prospective cohort. Two sample-pairs could not be compared due to lack of detectable SCAAs (patient P38) and suboptimal quality of analysis (patient P18) and were excluded from the comparison (Supplementary Table 5). Shared SCAAs were observed in 73% (11/15) of patients from WES data. Private mutations were detected in 33% (5/15) and 67% (10/15) of plasma and tissue DNA samples, respectively (Table 2 and Supplementary Table 5).

**Table 2:** Selected cancer-associated alterations (SCAAs) identified by WES in plasma and tissue DNA

Patient ID (cohort)	Cancer subtype	Shared SCAAs	SCAAs in cfDNA	SCAAs in tissue
P1 (2)	*Endometrial*	*ATM, ARID1A, AKT2*	*ATM, ARID1A, AKT2*	*ATM, ARID1A, AKT2, RAD51C*
P2 (2)	*Ovarian*	*TP53*	*TP53, FGF10*	*TP53*
P4 (2)	*Head and neck*	*TP53, RAD21*	*TP53, RAD21*	*TP53, RAD21, PIK3CA, CDKN2A, RET*
P6 (2)	*Pancreatic*	*KRAS, TP53*	*KRAS, TP53, FANCD2*	*KRAS, TP53, ATR x2*
P8 (2)	*Testicular*	*SMARCA5, RAB14*	*SMARCA5, RAB14, MYO6*	*SMARCA5, RAB14, POLR3A*
P9 (1)	*Prostate*	*TP53*	*TP53*	*TP53*
P13 (1)^F^	*Prostate*	*PTEN*	*PTEN, ATR*	*PTEN*
P23 (1)^F^	*Lung (NSCLC)*	*KRAS*	*KRAS, CHEK1*	*KRAS*
P37 (3)	*Breast*	*PIK3CA x2*	*PIK3CA x2*	*PIK3CA x2, TP53*
P40 (3)	*Colorectal*	*TP53*	*TP53*	*TP53, MET*
P41 (3)	*Bile duct*	*IDH1, ATR*	*IDH1, ATR*	*IDH1, ATR*

Alterations identified by whole exome sequencing (WES) in patients with SCAAs identified in plasma and tissue DNA (*N*= 11). Patients where mutations were only identified in either cfDNA or tissue DNA are shown in Supplementary Table 5.

^F^ FFPE material used for WES.

## DISCUSSION

In this study, we show that genomic tumor profiling of cfDNA was feasible in all 44 patients compared to only 25% of patients who were eligible for re-biopsy. Plasma cfDNA profiling thus represents a minimally-invasive alternative when tissue biopsies are not available. Especially, within the group of patients who cannot be biopsied, the benefit of applying cfDNA into cancer diagnostics is undoubtable. We successfully detected SCAAs in cfDNA in 8/9 patients in Cohort 1 primarily being NSCLC and prostate cancers with inaccessible thoracic and bone metastases, respectively. In all prospective prostate cancer samples, a SCAA was identified mainly being amplification of the *AR* gene which is a well-known resistance mechanism in 50% of castration-resistant prostate cancers [20, 21]. In these cancer types, the use of cfDNA for tumor profiling is of great importance as a growing number of targeted therapies and clinical trials are available for different molecular subtypes of prostate and lung cancers [22–24]. None of the 24 prospectively analyzed patients had tumor alterations identified in cfDNA that were actionable by an open clinical trial or off-label program at our institution at the time of analysis. This was in line with the general CoPPO cohort in which only 20% of patients (101/500 biopsied patients) received treatment based on tumor tissue profiling [25].

Despite a small sample size, this study indicates, that some cancer types might be more suitable for cfDNA profiling than others. Surprisingly, only 2/8 breast cancers had a SCAA identified by WES with mutation frequencies around 5%. None of the samples showed a positive finding on OncoScan, despite the high prevalence of SCNAs in breast cancers, often involving deletion of *PIK3CA* or amplification of *ERBB2*[26, 27]. These results could reflect a low fraction of plasma ctDNA in breast cancer patients that might be explained by the effect of active treatment at the time of plasma collection possibly increasing the level of normal cfDNA [28]. This was shown in a recent study of 210 patients with NSCLC, where ctDNA was detected in 43% of patients receiving systemic therapy at the time of plasma sampling and in 75% of patient not in therapy [29]. Moreover, the low ctDNA detection might also be related to a small tumor burden known to correlate with both cfDNA and ctDNA levels [30, 31]. We have not correlated our findings with tumor burden due to limited access to radiological data at the time of plasma collection. Furthermore, we did not include baseline RECIST measurements as these underestimated the actual tumor burden due to exclusion of non-target lesions [32].

Analysis of cfDNA immediately following failed biopsy, markedly decreased the time to completed genomic reports (median 29 vs 60 days), a key factor for timely allocation of treatment in the Phase1 setting. Even though, the time from failed biopsy until re-biopsy could be improved from the median 31 days observed here, cfDNA analysis would still be superior with respect to turnaround time. Furthermore, in some clinical settings, validation of potential treatment targets might be performed thus increasing the turnaround time concerning both tissue and cfDNA samples.

Despite all the advantages of cfDNA profiling, biopsy material remains the method of choice. The most obvious restriction of using cfDNA for genomic tumor profiling is the low tumor fraction (0.01–10%) in cfDNA [33, 34], in combination with the limited sensitivity of the current methods. OncoScan analysis only identified SCAAs in 13/44 patients most likely due to cfDNA input levels below the recommended 80 ng. In this small feasibility study, we could not assign any correlation between total cfDNA levels and the identification of ctDNA by either method (*P*>0.05). However, increased cfDNA levels might reflect a high degree of normal tissue destruction potentially diluting the ctDNA signal leading to false negative ctDNA results. In addition, the highly fragmented nature of cfDNA could also affect method efficacy. Solutions adjusted to small fragment DNA sizes are being developed [35], together with SCNA assays optimized for cfDNA [17]. Furthermore, it has been shown that increasing the cfDNA input to > 20 ng for WES analysis improved the detection of ctDNA [19].

Another challenge of using cfDNA for oncological diagnostics, is the intrapersonal tumor evolution and hereby, a high degree of tumor heterogeneity. In this study, 65% of the SCAAs identified by WES was shared between plasma and tissue and private mutations were detected in 33% and 67% of plasma and tissue, respectively. However, large comparative studies are needed to assess the concordance between tumor and plasma biopsies and ongoing studies of postmortem samples like the PEACE study (NCT03004755) might help clarifying this aspect. Alterations in cfDNA have been suggested to represent clonal alterations rather than subclonal [18], possibly explaining the lower number of SCAAs private to cfDNA compared to tissue observed here. The lack of complete concordance between cfDNA and tissue likely reflects: 1) That the clonal nature of the tumor is not captured by a single biopsy; 2) That some tumor clones shed little DNA, or the frequency of the alteration was below the cut-off of 5%; Or 3) that the tumor evolves over time and in response to therapy, mainly important for the FFPE samples, representing archival material potentially years older than the cfDNA sample.

To our knowledge, this is the first study to test WES and SCNA analysis by OncoScan of cfDNA in a prospective trial. Previous studies have successfully used cfDNA for genomic profiling, but these studies have included only few patients [36] or not been clinically relevant due to the high level of sequencing coverage and costs [37]. Our feasibility study was limited in sample size and heterogeneity of cancer types. Furthermore, we tested only WES and OncoScan as these methods were available in our clinical setting. Indications related to the effect of cancer type and active therapy on cfDNA profiling needs further validation in larger and uniform cohorts.

In conclusion, genomic tumor profiling using plasma cfDNA constitutes an alternative when tissue biopsies are unavailable. Especially, WES identified SCAAs and potential treatment targets whereas SCNA analysis by OncoScan needs optimization for cfDNA. Advanced prostate and lung cancer patients can particularly benefit from cfDNA profiling contrary to breast cancers, where preselection based on treatment status should be considered. Finally, more SCAAs were identified in fresh tumor tissue highlighting that tissue is still the preferable material for genomic profiling due to the higher density of tumor DNA.

## MATERIALS AND METHODS

### Patients

Patients with metastatic solid tumors were recruited to the Phase I Unit at Rigshospitalet, Department of Oncology, Copenhagen University, as part of the Copenhagen Prospective Personalized Oncology study (CoPPO) project (NCT02290522)[25]. The study was conducted in accordance with the Declaration of Helsinki and written informed consent was obtained for all patients (Danish Ethical Committee, file number: 1300530). We included three cohorts: 1) A prospective cohort (Cohort 1; *N*=9) including patients with tumors inaccessible for biopsy due to location e.g. bone metastasis only, inaccessible thoracic lesions etc.; 2) A prospective cohort (Cohort 2; *N*=15) including patients whose initial tissue biopsy failed the genomic analyses due to low levels of tumor cells (<10%); 3) A retrospective cohort (Cohort 3; *N*=20), consisting of patients whose initial biopsy failed and was unusable for genomic profiling. The inclusion period for Cohort 1 and 2 was from January to 1^st^ of August 2018.

### DNA collection and purification

Two blood samples were collected from each patient, one for germline DNA (gDNA) analysis as described for the CoPPO study [25] and one for cfDNA analysis. Peripheral blood for cfDNA analysis was collected in cell-stabilizing BCT-tubes (Streck Laboratories) as previously described [38] and extracted from 4 ml plasma using the QIAsymphony Circulating DNA Kit (Qiagen) according to the manufacturer's instructions using an elution volume of 60 μl. Extracted cfDNA was quantified using a dsDNA HS Assay Kit on a Qubit Fluorometer (Thermo Fisher Scientific) and subsequently stored at -20°C until further use. For patients with a successful re-biopsy, fresh tumor tissue was collected in RNA-later as part of the CoPPO project. Archival FFPE tissue was included if available and DNA was extracted using the Gene Read DNA FFPE Kit (Qiagen).

### Genomic profiling

DNA libraries were prepared from 10 ng cfDNA or FFPE-DNA using the NEBNext Ultra II protocol (New England Biolabs) and hybridized using the MedExome capture panel (Roche) or Agilent SureSelect system, respectively. DNA libraries from gDNA or fresh tissue re-biopsies were generated from 500 ng DNA using SureSelect (Agilent). All DNA libraries enriched for exonic sequences were then quantified and quality-controlled using a Qubit HS flourometer and the TapeStation 4200 High Sensitivity assay (Agilent). Finally, all libraries were sequenced as paired-end on the NextSeq or HiSeq sequencer (Illumina). Plasma cfDNA libraries were sequenced with an average coverage >100x (Supplementary Figure 2).

Sequencing reads were mapped to the hg19/GRCh37 reference genome using the CLC Workbench v.3.5.4. Tumor-specific variants were identified by excluding germline variants and requiring a variant to have 1) ≥ 10x coverage; 2) forward/reverse balance ≥ 0.1; (3) tumor variant frequency ≥ 5%. Variants were identified using the Ingenuity Variant Analysis Software (Qiagen) and selected cancer-associated alterations (SCAAs) were manually inspected and included in the final genomic reports comprising: 1) Previously reported cancer variants described in COSMIC [39], Ingenuity Knowledge database (Qiagen) or in published literature and 2) novel truncating alterations in tumor suppressor genes or missense alterations in known oncogenes predicted by *in silico* analysis [40] to be affecting protein function. The SCAA definition thus included class I to III variants according to the Tier classification [41]. The analysis excluded low quality and common variants (>1% in the Exome Aggregation Consortium database (ExAC), 1000 Genomes Project or NHLBI Exome sequencing project (ESP)) and included non-synonymous alterations as well as splice site alterations +/- 2bp from exon/intron boundaries.

Somatic copy number alterations (SCNA) were analysed using the OncoScan CNV Plus assay (ThermoFisher Scientific) with minimum input of 5 ng cfDNA. The data was visually inspected and analysed using the Nexus Software v 8.0 (BioDiscovery). The analysis of SCNAs focused on detection of amplification (copy number >5), bi-allelic losses, deletions, and loss of heterozygosity (LOH). On fresh tissue re-biopsies, SCNAs were analyzed using CytoScanHD assay (Affymetrix) with DNA input of 250 ng according to the manufactures instructions. Sensitivity of the applied methods were 5% and 20–30% for WES and OncoScan, respectively. Hence a negative report does not necessarily indicate lack of tumor specific alterations or ctDNA.

### Statistical analyses

A t-test was used to test, whether cfDNA concentration or DNA input affected the results from OncoScan analysis. Due to small sample sizes, Fishers-exact test was performed to compare the number of breast cancer patients harboring a SCAA to those with no SCAA. A *P*-value <0.05 was considered statistically significant. Furthermore, descriptive statistics were used, including medians, ranges, and frequencies. All statistical analyses were done in R (version 0.99.903).

## SUPPLEMENTARY MATERIALS FIGURES AND TABLES





## Abbreviations

cfDNA: cell-free DNA
ctDNA: Circulating tumor DNA
WES: whole exome sequencing
WGS: whole genome sequencing
SCNAs: somatic copy number alterations
FFPE: formalin-fixed paraffin-embedded
NSCLC: non-small cell lung cancers
NGS: next generation sequencing
CoPPO: Copenhagen Prospective Personalized Oncology study
gDNA: germline DNA
SCAA: selected cancer-associated alterations
ExAC: Exome Aggregation Consortium database
ESP: Exome sequencing project
IQR: Inter quartile range.
