# Supplementary material for: Application of cell-free DNA for genomic tumor profiling: a feasibility study
Source: Oncotarget. 2019 Feb 15;10(14):1388–98. doi: 10.18632/oncotarget.26642 (PMC6402712; doi:10.18632/oncotarget.26642)
Supplement: Supplementary file 1 [file oncotarget-10-1388-s001.pdf]

Application of cell-free DNA for genomic tumor profiling: a feasibility study

SUPPLEMENTARY MATERIALS

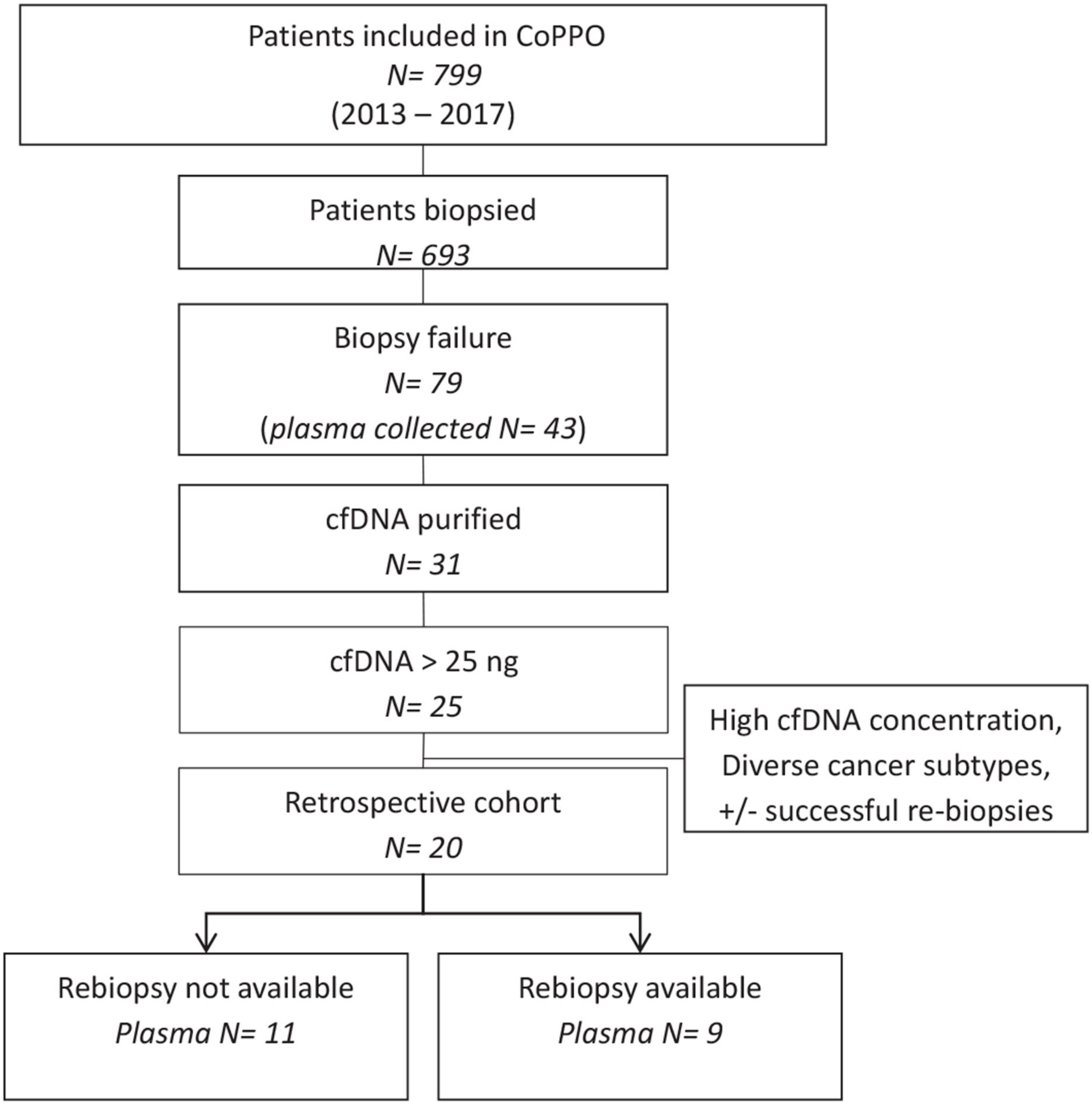

Supplementary Figure 1: Selection criteria for the retrospective cohort. Patients were selected from the CoPPO cohort included in the period 2013 - 2017. In total, 799 patients were included and 693 had a tissue biopsy performed. In 79 cases the tissue biopsy was not usable for genomic profiling primarily due to low tumor cell content (<10%). Plasma cfDNA had been collected in 43 cases and 25 samples had sufficient cfDNA (> 25 ng) for WES and SCNA analysis (OncoScan). We selected the 20 samples with highest cfDNA concentrations, diverse cancer types, and patients with/without a successful re-biopsy to compare tissue and plasma DNA profiles.

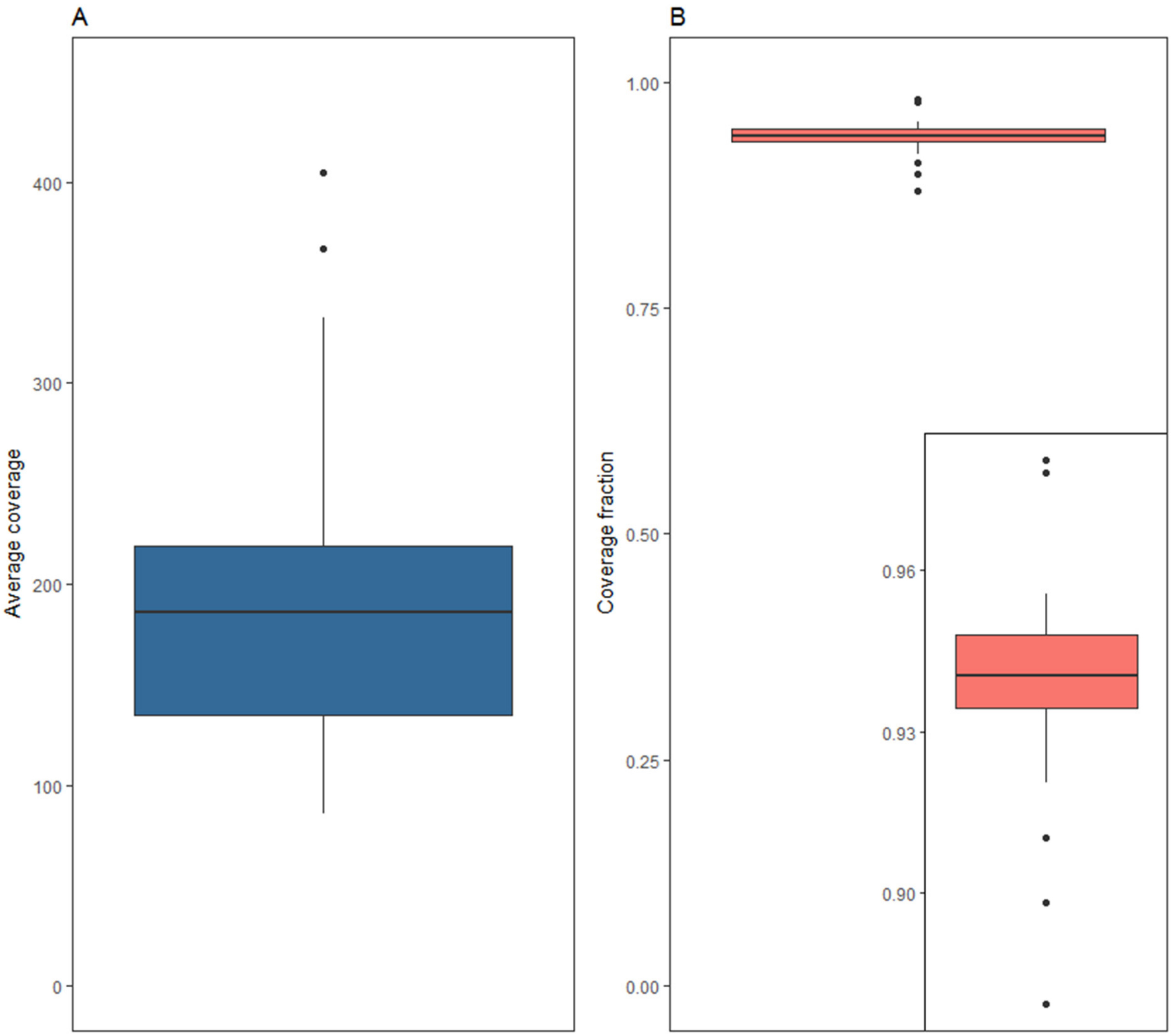

Supplementary Figure 2: Whole exome sequencing coverage. (A) Shows the distribution of the average coverage from the 44 WES analyses. The median overall average coverage was 186x (interquartile range IQR=273.50; Q1=90.80; Q3=364.30). **(B)** Illustrates the fraction of the exome covered by ≥10x with a median of 94% (interquartile range IQR=0.0135; Q1=0.9344; Q3=0.9479). Detailed information is provided in Supplementary Table 2.

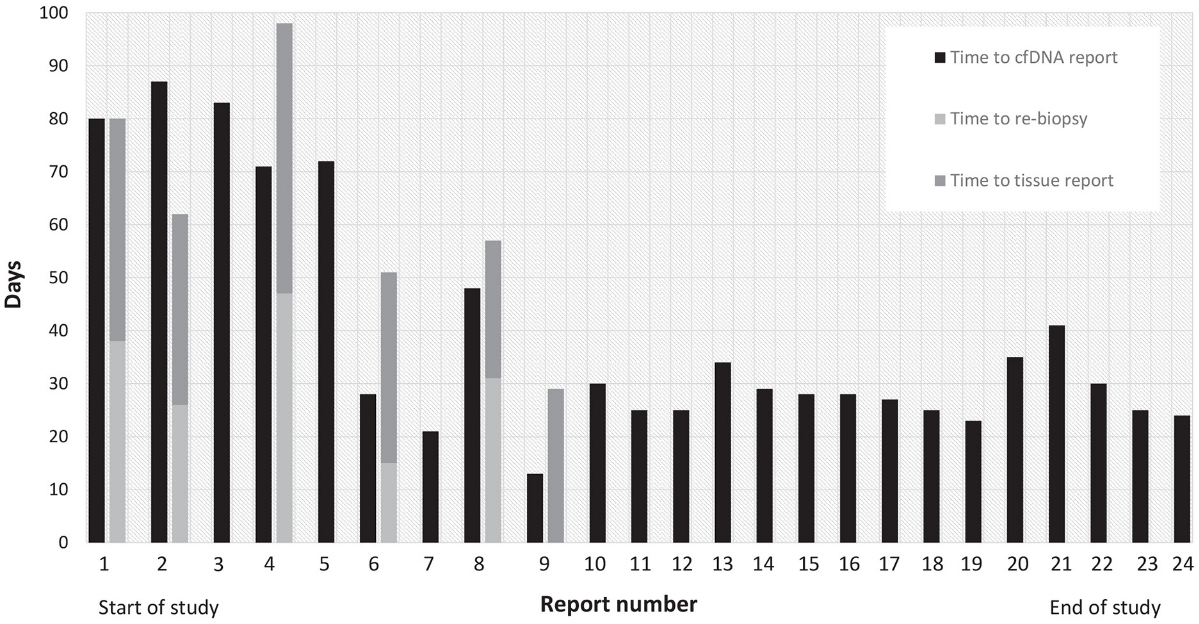

Supplementary Figure 3: Turnaround time for prospective genomic profiling of cfDNA and tissue re-biopsies. The turnaround time for cfDNA analyses is indicated in black representing the time from failed biopsy until genomic report. Turnaround time for tissue biopsies are indicated for 6 patients (report no. 1, 2, 4, 6, 8, 9) with an additional column showing the time from failed biopsy until second biopsy (light grey) and the time from second biopsy until a complete genomic report (dark grey). Report no. 9 represents a patient where no biopsy could be obtained at inclusion, but the patient later presented with an accessible lesion and a biopsy was obtained. The x-axis shows the cfDNA report number corresponding to the 24 prospectively profiled patients (Supplementary Table 1). Report no. 9, 11, 13, 16, 17, 19, 20, 22, 23 represented patients in Cohort 1. The median turnaround times from failed biopsies to completed genomic reports were 29 days (range 13-87 days) and 60 days (range 29-98) for cfDNA and tissue, respectively.

Supplementary Table 1: Tumor information and turnaround time for genomic reports across cohorts (*N*= 44)

The turnaround time includes the time from failed biopsy or no biopsy until the genomic report was completed. Turnaround time is only reported for patients prospectively included in the study. Indication of treatment and location of metastatic sites at the time of blood collection were included. Identification of ctDNA by WES and/or SCNA analysis (OncoScan) is indicated by a +/- SCAA. [R] This report time included analysis of a second plasma sample, as the first 4 ml plasma did not contain >10ng cfDNA. [B] For this patient no biopsy could be obtained at inclusion, but the patient later presented with an accessible lesion and was biopsied. Abbreviations: WES: Whole exome sequencing, SCNA: Somatic copy number alteration; SCAA: Selected cancer-associated alterations, LN: Lymph node, FFPE: Formalin-fixed paraffin embedded, NSCLC: non-small cell lung cancer, F: Female, M: Male, Y: Yes, N: No, NA: Data not available.

See Supplementary File 1

Supplementary Table 2: Whole exome sequencing and OncoScan input and quality across cohorts (*N*= 44).

The average coverage from WES and the percentage of the exome covered by at least 10x is reported for each patient (*N*=44). The median average coverage and >10x coverage was 186x and 94%, respectively. The cfDNA concentration in nanogram per ml plasma measured by the Qubit dsDNA HS assay is also included together with the amount of cfDNA used for OncoScan analyses. Quality parameters from OncoScan analysis included MAPD (Median of the Absolute Values of all Pairwise Differences) and ndWavinessSD (Normal Diploid Waviness Standard Deviation). MAPD is a global measure of the variation of all microarray probes across the genome. It represents the median of the distribution of changes in log2 ratio between adjacent probes. NdWavinessSD is a global measure of variation of microarray probes that is insensitive to short-range variation and focuses on long-range variation. ndWavinessSD is computed on normal diploid markers. *No analysis* indicates the samples where no OncoScan analysis was performed due to limited cfDNA material. Identification of ctDNA by WES and/or SCNA analysis (OncoScan) is indicated by a +/- SCAA. Abbreviations: WES: Whole exome sequencing, SCNA: Somatic copy number alteration.

See Supplementary File 1

Supplementary Table 3: Selected cancer-associated alterations (SCAAs) identified in cfDNA using WES (*N*=44)

The variant frequency (%) in plasma cfDNA and sequencing coverage (x) is reported for each selected cancer-associated alteration (SCAA) identified by WES. A representative COSMIC identifier (ID) is provided for variants previously reported in cancer. If no COSMIC ID existed, the dbSNP or ClinVar ID was included. *: stop codon. [IS]: *In silico* analysis using the integrated software Alamut version 2.7, indicate that this mutation was likely to be damaging to the protein function (http://www.interactive-biosoftware.com).

See Supplementary File 1

Supplementary Table 4: Selected cancer-associated somatic copy number alterations in cfDNA (OncoScan, *N*= 44).

Results from OncoScan analysis (*N*=40) including amplifications and deletions of selected cancer-associated alterations (SCAA). Subchromosomal gains and deletions as well as chromosomal numerical changes were also reported. Indication of homologous recombination deficiency (HRD) was based on the number of LOH segments larger than 15MB. Silent profiles: No genomic alterations could be detected, and samples showed euploid chromosomes corresponding to the normal tissue profile. Failed (suboptimal quality): The results from OncoScan could not be processed due to the suboptimal sample quality (positive and negative controls run in parallel). Abbreviations: Chr: Chromosomal, LOH: Loss of heterozygosity.

See Supplementary File 1

Supplementary Table 5. Comparison of genomic alterations identified in matched plasma and tissue DNA (*N*= 17)

Selected cancer-associated alterations (SCAAs) identified in plasma and tissue DNA for 17 patients across cohorts. Results from both WES and OncoScan are indicated together with analyses performed on fresh tumor tissue biopsies or FFPE tissue[F]. The SNP array analyses of SCNAs differed between materials; OncoScan was used for cfDNA and CytoScanHD was used for tumor tissue DNA. The difference in SCAAs between the two materials are highlighted in bold. Abbreviations: HRD: Homologous recombination deficiency, Chr: Chromosome, SNP: Single nucleotide polymorphism.

See Supplementary File 1
